# Effectiveness of an Artificial Intelligence-Assisted App for Improving Eating Behaviors: Mixed Methods Evaluation

**DOI:** 10.2196/46036

**Published:** 2024-05-07

**Authors:** Han Shi Jocelyn Chew, Nicholas WS Chew, Shaun Seh Ern Loong, Su Lin Lim, Wai San Wilson Tam, Yip Han Chin, Ariana M Chao, Georgios K Dimitriadish, Yujia Gao, Jimmy Bok Yan So, Asim Shabbir, Kee Yuan Ngiam

**Affiliations:** 1 Alice Lee Centre for Nursing Studies Yong Loo Lin School of Medicine National University of Singapore Singapore Singapore; 2 Department of Cardiology National University Hospital Singapore Singapore; 3 Yong Loo Lin School of Medicine National University of Singapore Singapore Singapore; 4 Department of Dietetics National University Hospital Singapore Singapore; 5 School of Nursing Johns Hopkins University Baltimore, MD United States; 6 Department of Endocrinology ASO/EASO COM King's College Hospital NHS Foundation Trust London United Kingdom; 7 Division of Hepatobiliary & Pancreatic Surgery Department of Surgery National University Hospital Singapore Singapore; 8 Division of General Surgery (Upper Gastrointestinal Surgery) Department of Surgery National University Hospital Singapore Singapore; 9 Division of Thyroid & Endocrine Surgery Department of Surgery National University Hospital Singapore Singapore

**Keywords:** artificial intelligence, chatbot, chatbots, weight, overweight, eating, food, weight loss, mHealth, mobile health, app, apps, applications, self-regulation, self-monitoring, anxiety, depression, consideration of future consequences, mental health, conversational agent, conversational agents, eating behavior, healthy eating, food consumption, obese, obesity, diet, dietary

## Abstract

**Background:**

A plethora of weight management apps are available, but many individuals, especially those living with overweight and obesity, still struggle to achieve adequate weight loss. An emerging area in weight management is the support for one’s self-regulation over momentary eating impulses.

**Objective:**

This study aims to examine the feasibility and effectiveness of a novel artificial intelligence–assisted weight management app in improving eating behaviors in a Southeast Asian cohort.

**Methods:**

A single-group pretest-posttest study was conducted. Participants completed the 1-week run-in period of a 12-week app-based weight management program called the Eating Trigger-Response Inhibition Program (eTRIP). This self-monitoring system was built upon 3 main components, namely, (1) chatbot-based check-ins on eating lapse triggers, (2) food-based computer vision image recognition (system built based on local food items), and (3) automated time-based nudges and meal stopwatch. At every mealtime, participants were prompted to take a picture of their food items, which were identified by a computer vision image recognition technology, thereby triggering a set of chatbot-initiated questions on eating triggers such as who the users were eating with. Paired 2-sided *t* tests were used to compare the differences in the psychobehavioral constructs before and after the 7-day program, including overeating habits, snacking habits, consideration of future consequences, self-regulation of eating behaviors, anxiety, depression, and physical activity. Qualitative feedback were analyzed by content analysis according to 4 steps, namely, decontextualization, recontextualization, categorization, and compilation.

**Results:**

The mean age, self-reported BMI, and waist circumference of the participants were 31.25 (SD 9.98) years, 28.86 (SD 7.02) kg/m^2^, and 92.60 (SD 18.24) cm, respectively. There were significant improvements in all the 7 psychobehavioral constructs, except for anxiety. After adjusting for multiple comparisons, statistically significant improvements were found for overeating habits (mean –0.32, SD 1.16; *P*<.001), snacking habits (mean –0.22, SD 1.12; *P*<.002), self-regulation of eating behavior (mean 0.08, SD 0.49; *P*=.007), depression (mean –0.12, SD 0.74; *P*=.007), and physical activity (mean 1288.60, SD 3055.20 metabolic equivalent task-min/day; *P*<.001). Forty-one participants reported skipping at least 1 meal (ie, breakfast, lunch, or dinner), summing to 578 (67.1%) of the 862 meals skipped. Of the 230 participants, 80 (34.8%) provided textual feedback that indicated satisfactory user experience with eTRIP. Four themes emerged, namely, (1) becoming more mindful of self-monitoring, (2) personalized reminders with prompts and chatbot, (3) food logging with image recognition, and (4) engaging with a simple, easy, and appealing user interface. The attrition rate was 8.4% (21/251).

**Conclusions:**

eTRIP is a feasible and effective weight management program to be tested in a larger population for its effectiveness and sustainability as a personalized weight management program for people with overweight and obesity.

**Trial Registration:**

ClinicalTrials.gov NCT04833803; https://classic.clinicaltrials.gov/ct2/show/NCT04833803

## Introduction

Overweight and obesity remain a public health concern that affects slightly more than half of the global adult population [1]. Across 52 Organization for Economic Co-operation and Development, Group of Twenty, and European Union 28 countries, treating conditions related to overweight and obesity costs US $425 billion per year, based on purchasing power parity. Each US dollar used to prevent obesity results in a 6-fold return in economic benefits [2]. Strategies for maintaining a healthy weight range from policy mandates on nutritional food labeling [3] to clinical treatments focused on lifestyle modifications, pharmacotherapy, and bariatric surgery [4]. However, the effectiveness of such strategies is limited by insurance coverage [5] and challenges with weight loss maintenance [6-9]. Some participants have been reported to regain up to 100% of their initial weight loss within 5 years [9,10].

With the rapid digitalization and smartphone penetration worldwide, weight loss apps have been gaining popularity, as they help overcome the temporospatial challenges of in-person weight loss programs [11]. For instance, participants enrolled in conventional weight management programs typically attend multiple face-to-face sessions at designated facilities, which could be burdensome and inconvenient as one needs to schedule appointments and travel to the facility that may be beyond one’s usual mobility pattern. Moreover, such programs are resource-intensive, requiring a multidisciplinary team of trained health care professionals (eg, physicians, dietitians, physiotherapists, nurses), infrastructure (eg, counselling room), and equipment (eg, weighing scale, stadiometer) to maintain. Well-known apps that support weight loss in the market include MyFitnessPal [12], MyPlate Calorie Tracker [13], and Fitbit [14]. In Singapore, Healthy 365 [15] is available for the public, while nBuddy [16] is used for the clinical population. These apps mostly focus on calorie tracking, health status tracking, and progress monitoring. Increasingly, apps are enhanced with features that allow intuitive synchronization of health metrics across apps to provide a more holistic progress monitoring experience. With a fee, some apps even match users to a health coach who would provide personalized weight management plans to support weight loss. However, there is a need for apps that include monitoring and support for one’s self-regulation over momentary eating impulses, which are often triggered and influenced by dietary lapse triggers such as visual food cues, eating out, negative affect, and sleep deprivation [17-20]. Self-regulation of eating behaviors during weight loss treatment commonly includes portion control, increasing fruit and vegetable consumption, reducing unhealthy food (sugar-sweetened beverages and high-fat food items) consumption, and reducing overall caloric consumption [17]. Therefore, we aimed to examine the feasibility and effectiveness of a novel artificial intelligence (AI)-assisted weight management app on improving eating behaviors and to explore the mechanism by which this app influences eating behaviors, as hypothesized in our earlier work [21].

## Methods

### Study Design

A single-group pretest-posttest study was conducted and reported according to the TREND (Transparent Reporting of Evaluations with Nonrandomized Designs) checklist (Multimedia Appendix 1) [22]. Despite the limitations of the study design, it was deemed the most appropriate and feasible experimental study design for a preliminary understanding of the usability, acceptability, and effectiveness of the app [23].

### Participant Recruitment

Participants older than 21 years with BMI ≥23 kg/m^2^ and not undergoing a commercial weight loss program were recruited from January 2022 to October 2022 through social media platforms and physical recruitment at a local tertiary hospital’s specialist weight management clinic in Singapore. Using G*Power (version 3.1.9.7) [24], to detect a small effect size of 0.2 at .05 significance level and 80% power while accounting for an attrition rate of 20%, 248 participants are required. To be conservative, 250 participants were recruited.

### Intervention

Immediately after completing the pretest questionnaire, participants were onboarded to the Eating Trigger-Response Inhibition Program (eTRIP) app by a trained research assistant to complete the 1-week run-in of the program. During the onboarding, participants were invited to enter their anthropometric details, desired weight loss goals, and motivation. They were also encouraged to personalize certain app functions such as the timing of the check-in prompts and preferred name for interaction with a chatbot. At every mealtime (at least 3 times a day), the participants were prompted to take a picture of their food, which was immediately recognized by a food-based computer vision image recognition technology, which then triggered a set of chatbot-initiated questions on eating triggers (eg, how they are feeling). These questions were developed based on our past work on eating behaviors [25-30]. Participants were able to view their image-based food log and eating habits on a dashboard, reflect upon their eating habits throughout the day, and set their goals and action plans for the next day. On the 8th day, all participants’ user accounts were locked, and they were unable to make any changes but were able to still view their check-in logs. Participants could also provide feedback on the app by filling out the comments section in one of the app pages. Participants were reimbursed SGD 25 (SGD 1=US $0.74) for completing this program.

### eTRIP App

The eTRIP app was developed as a 12-week AI-assisted, app-based, self-regulation program targeted at improving weight loss through healthy eating. eTRIP was developed largely based on a modified temporal self-regulation theory [31,32], behavioral change taxonomy [33], and our previous work on healthy eating and weight loss [27-29,34,35]. This includes studies on people with overweight and obesity in the areas of personal motivators, self-regulation facilitators, and barriers [27]; the potential of AI, apps, and chatbots in improving weight loss [6,25,29]; perceptions and needs of AI to increase its adoption in weight management [26]; and the essential elements of a weight loss app [28]. The development of eTRIP was split into 2 phases: (1) development of an AI-assisted self-monitoring system and (2) development of an AI-assisted behavioral nudging system. In this paper, we report the feasibility and effectiveness of an AI-assisted self-monitoring system after a 1-week run-in. The self-monitoring system is built upon 3 main components, namely, (1) chatbot-based check-ins on eating lapse triggers, (2) food-based computer vision image recognition (system built based on local food items), and (3) automated time-based nudges and meal stopwatch.

### Outcomes

All participants completed the same self-report questionnaire before and after the 1-week run-in of the app, which reflected their sociodemographic profile, BMI, waist circumference, intention to improve eating behaviors, habits of overeating (Self-Report Habit Index) [36], habits of snacking [36], consideration of future consequences (Consideration of Future Consequences Scale-6 items) [37], self-regulation of eating behavior (Self-Regulation of Eating Behavior Questionnaire) [38], physical activity (International Physical Activity Questionnaire-Short Form) [39], anxiety symptoms (Generalized Anxiety Disorder-2 items) [40], and depressive symptoms (Patient Health Questionnaire-2 items) [41]. Details are reported in Multimedia Appendix 2. The primary outcomes were overeating habits, snacking habits, immediate thinking, self-regulation of eating habits, depression, anxiety, and physical activity. The secondary outcomes were their subscale scores.

### Data Analysis

SPSS statistical software (version 27; IBM Corp) [42] was used for the analyses. The baseline characteristics of the participants were presented in mean (SD) and frequency (%). Paired 2-sided *t* tests were used to compare the differences in the psychobehavioral constructs before and after the 7-day program, including overeating habits, snacking habits, consideration of future consequences, self-regulation of eating behaviors, anxiety, depression, and physical activity. To account for the increased risk of a type 1 error due to multiple comparisons [43], the Bonferroni-corrected significant level was set to *P*≤.007. Qualitative feedback were analyzed using content analysis according to 4 steps, namely, decontextualization, recontextualization, categorization, and compilation [44]. Feedback was first consolidated verbatim and read iteratively by 2 coders (Nagadarshini Nicole Rajasegaran and HSJC). The verbatim feedback was then analyzed independently by 2 reviewers into meaning units. Meaning units were then reconstituted, categorized, and reported as themes and subthemes.

### Ethics Approval

This single-group pretest-posttest study was approved by the National Healthcare Group Domain Specific Review Board (ref 2020/01439), registered with the ClinicalTrials.gov (ref NCT04833803) on April 6, 2021.

## Results

### Baseline Characteristics of the Participants

A total of 251 participants were enrolled in this study (Chew HSJ, unpublished data, 2023); 20 (7.9%) participants dropped out of the 1-week program due to the inability to perform check-ins every day. Among those who completed the program (n=231), 1 participant was removed from the analyses due to ineligibility. The mean age, self-reported BMI, and waist circumference of the participants was 31.25 (SD 9.98) years, 28.86 (SD 7.02) kg/m^2^, and 92.6 (SD 18.24) cm, respectively (Table 1). Approximately 47.8% (111/230) of the participants were males, indicating a good mix of participants from both sexes, and most of the participants were single (169/230, 73.6%), Chinese (181/230, 78.7%), and had a university education (148/230, 64.1%).

**Table 1:** Baseline characteristics of the participants who completed and who dropped out from the 1-week program.

Characteristics			Completed (n=230)		Dropped out (n=20)
Age (years), mean (SD)			31.25 (9.98)		36.25 (10.29)
Young adults (21-35 years), n (%)			168 (73)		11 (55)
Middle-aged adults (36-64 years), n (%)			62 (27)		9 (45)
**Sex, n (%)**					
	Males	110 (48)		9 (45)	
	Females	120 (52)		11 (55)	
**Marital status, n (%)**					
	Single	169 (74)		7 (35)	
	Married	59 (26)		12 (60)	
	Divorced	2 (1)		1 (5)	
**Race, n (%)**					
	Chinese	181 (79)		7 (35)	
	Malay	19 (8)		7 (35)	
	Indian	24 (10)		5 (25)	
	Others	6 (3)		1 (5)	
**Religion, n (%)**					
	Buddhism	75 (33)		2 (10)	
	Christianity	57 (25)		3 (15)	
	Hinduism	16 (7)		2 (10)	
	Islam	25 (11)		10 (50)	
	Free thinker	43 (19)		3 (15)	
	Others	14 (6)		0 (0)	
**Highest education level, n (%)**					
	Primary school	2 (1)		0 (0)	
	Secondary school	9 (4)		2 (10)	
	Institute of Technical Education/polytechnic/junior college	71 (31)		6 (30)	
	University	148 (64)		12 (60)	
**Per capita household income (SGD/month)^a^, n (%)**					
	<1000	21 (9)		2 (10)	
	1000-3000	47 (20)		6 (30)	
	3001-5000	57 (25)		3 (15)	
	5001-10,000	79 (34)		6 (30)	
	>10,000	26 (11)		3 (15)	
**Residential region, n (%)**					
	Central	41 (18)		3 (15)	
	East	17 (7)		3 (15)	
	North	22 (10)		1 (5)	
	Northeast	36 (16)		1 (5)	
	West	76 (33)		5 (25)	
**Smoking, n (%)**					
	No	220 (96)		18 (90)	
	Yes	10 (4)		2 (10)	
**Employment, n (%)**					
	Part-time	51 (22)		2 (10)	
	Full-time	171 (74)		18 (90)	
	Retired	9 (4)		0 (0)	
BMI (kg/m^2^), mean (SD)			28.86 (7.02)		33.73 (8.21)
**Weight status, n (%)**					
	Overweight (23.0-27.4 kg/m^2^, moderate risk)	142 (62)		4 (20)	
	Class I (27.5-32.4 kg/m^2^, high risk)	41 (18)		6 (30)	
	Class II (32.5-37.4 kg/m^2^, very high risk)	16 (7)		4 (20)	
	Class III (≥37.4 kg/m^2^, very high risk)	31 (14)		6 (30)	
BMI (*z* score), mean (SD)			–0.05 (0.97)		0.62 (1.14)
Waist circumference (cm), mean (SD)			92.60 (18.24)		102.75 (23.69)
Large waist circumference (male ≥90 cm; female ≥80 cm), n (%)			153 (67)		18 (90)
Waist circumference (*z* score), mean (SD)			–0.04 (0.97)		0.50 (1.26)
Generalized anxiety disorder-2 items score, mean (SD)			1.85 (1.63)		1.10 (0.85)
Potentially at risk cutoff (≥3), n (%)			61 (27)		0 (0)
Patient health questionnaire-2 items score, mean (SD)			1.56 (1.62)		1.3 (1.26)
Potentially at risk cutoff (≥3), n (%)			50 (22)		2 (10)

^a^SGD 1=US $0.74.

### Mean Baseline Scores on Each Outcome Variable

The mean baseline scores on each outcome variable of the participants who completed and who dropped out from the 1-week program were calculated (Table 2). As the dropout rate was only 8.4% (20/251), statistical comparisons between those who dropped out and those who completed the program was not necessary.

**Table 2:** Mean baseline scores on each outcome variable of the participants who completed and who dropped out from the 1-week program.

Characteristics			Completed (n=230)		Dropped out (n=20)
Intention to change eating behaviors, mean (SD)			5.71 (1.16)		6.00 (1.30)
**Overeating, mean (SD)**					
	SRHI^a^	4.28 (1.54)		4.11 (1.34)	
	Behavioral frequency	4.44 (1.63)		4.32 (1.56)	
	Automaticity	4.35 (1.60)		4.06 (1.46)	
	Self-identify	4.05 (1.59)		3.95 (1.3)	
**Snacking, mean (SD)**					
	SRHI	4.20 (1.47)		4.20 (1.08)	
	Behavioral frequency	4.46 (1.59)		4.83 (1.33)	
	Automaticity	4.03 (1.62)		3.85 (1.24)	
	Self-identify	4.10 (1.49)		3.91 (1.23)	
**CFCS-6^b^, mean (SD)**					
	Total	4.34 (1.11)		4.67 (1.08)	
	Immediate	4.30 (1.56)		4.00 (1.57)	
	Future	4.98 (1.11)		5.33 (0.86)	
**SREBQ^c^, mean (SD)**			2.86 (0.53)		2.89 (0.42)
	Low, n (%)	86 (37)		4 (20)	
	Moderate, n (%)	133 (58)		16 (80)	
	High, n (%)	11 (5)		0 (0)	
**IPAQ-SF^d^ (MET^e^-min/week), mean (SD)**			2230.76 (2503.66)^f^		1678.95 (3114.76)
	Low, n (%)	76 (35)		12 (60)	
	Moderate, n (%)	63 (29)		2 (10)	
	Vigorous, n (%)	79 (34)		6 (30)	
**Identifies the following foods as tempting, n (%)**					
	Chocolate	149 (65)		15 (75)	
	Crisps	160 (70)		12 (60)	
	Cake	151 (66)		16 (80)	
	Ice cream	141 (61)		10 (50)	
	Bread/toast	100 (44)		9 (45)	
	Fizzy drinks	54 (24)		5 (25)	
	Biscuits	58 (25)		2 (10)	
	Sweets	43 (19)		3 (15)	
	Popcorn	34 (15)		3 (15)	
	Pastries	96 (42)		6 (30)	
	Pizza	91 (40)		9 (45)	
	Fried foods	138 (60)		11 (55)	
	Chips	106 (46)		12 (60)	
	Nil	8 (4)		0 (0)	
	Others	40 (17)		1 (5)	

^a^SRHI: Self-Report Habit Index.

^b^CFCS-6: Consideration of Future Consequences Scale-6 items.

^c^SREBQ: Self-Regulation of Eating Behavior Questionnaire.

^d^IPAQ-SF: International Physical Activity Questionnaire-Short Form.

^e^MET: metabolic equivalent task.

^f^n=218.

### Pretest and Posttest Mean Differences

There were significant improvements in all the 7 psychobehavioral constructs, except for anxiety. After adjusting for multiple comparisons, there were only statistically significant improvements in the overeating habit, snacking habit, self-regulation of eating behavior, depression, and physical activity (Table 3). Forty-one participants reported skipping at least 1 meal (ie, breakfast, lunch, or dinner), summing to a total of 578 (67.1%) of the 862 meals skipped.

**Table 3:** Mean differences in each outcome before and after the 1-week program for completers (n=230).

Outcomes			Pretest, mean (SD)		Posttest, mean (SD)		Mean difference (SD)		*t* *(df)*		*P* value
**Overeating**											
	SRHI^a^	4.28 (1.54)		3.96 (0.10)		–0.32 (1.16)		–4.21 (458)		<.001^b^	
	Behavioral frequency	4.44 (1.63)		4.11 (1.61)		–0.33 (1.23)		–4.12 (458)		<.001^b^	
	Automaticity	4.36 (1.61)		4.00 (1.55)		–0.36 (1.31)		–4.23 (458)		<.001^b^	
	Self-identity	4.05 (1.59)		3.78 (1.52)		–0.27 (1.24)		–3.29 (458)		<.001^b^	
**Snacking**											
	SRHI	4.19 (1.48)		3.97 (1.45)		–0.22 (1.12)		–2.96 (458)		.002^b^	
	Behavioral frequency	4.46 (1.60)		4.20 (1.60)		–0.26 (1.25)		–3.09 (458)		<.001^b^	
	Automaticity	4.01 (1.62)		3.77 (1.54)		–0.24 (1.35)		–2.71 (458)		.004^b^	
	Self-identity	4.09 (1.50)		3.93 (1.47)		–0.16 (1.20)		–1.98 (458)		.02	
**CFCS-6^c^**											
	Total	4.34 (1.11)		4.45 (1.08)		0.10 (0.89)		1.75 (458)		.04	
	Immediate^d^	4.29 (1.57)		4.10 (1.55)		–0.18 (1.27)		–2.18 (458)		.015	
	Future^e^	4.98 (1.11)		5.00 (1.11)		0.02 (1.09)		0.30 (458)		.38	
SREBQ^f^			2.85 (0.53)		2.93 (0.51)		0.08 (0.49)		2.46 (458)		.007^b^
Anxiety			1.92 (0.81)		1.84 (0.87)		–0.08 (0.80)		–1.58 (458)		.06
Depression			1.79 (0.81)		1.67 (0.84)		–0.12 (0.74)		–2.46 (458)		.007^b^
**IPAQ-SF^g^**											
	Total (n=128)	2569.45 (2514.51)		3858.05 (3327.26)		1288.60 (3055.20)		4.77 (254)		<.001^b^	
	Vigorous (n=170)	939.53 (1279.50)		1121.18 (1314.25)		181.65 (1313.22)		1.80 (338)		.04	
	Moderate (n=146)	487.12 (855.41)		974.25 (1328.51)		487.12 (1379.22)		4.27 (290)		<.001^b^	
	Walk	1061.95 (1445.45)		1335.71 (1621.90)		273.76 (1649.22)		2.52 (458)		.006^b^	

^a^SRHI: Self-Report Habit Index.

^b^Significant at *P*<.007.

^c^CFCS-6: Consideration of Future Consequences Scale-6 items.

^d^Consideration of Future Consequences Scale-6 immediate subscale.

^e^Consideration of Future Consequences Scale-6 future subscale.

^f^SREBQ: Self-Regulation of Eating Behavior Questionnaire.

^g^IPAQ-SF: International Physical Activity Questionnaire-Short Form.

### User Engagement

Among those who completed the program, 97% (46,867/48,316) chatbot-based questions were completed. As participants were given the option to add additional check-ins for snacks, the percentage of completed check-ins could not be accurately computed.

### Qualitative Feedback

Of the 230 participants, 80 (34.8%) provided textual feedback that indicated satisfactory experience with eTRIP. Four themes emerged, namely, (1) becoming more mindful of self-monitoring, (2) personalized reminders with prompts and chatbot, (3) food logging with image recognition, and (4) engaging with a simple, easy, and appealing user interface.

#### Becoming More Mindful of Self-Monitoring

By checking in with the app for every meal, the participants mentioned being more aware of their unhealthy eating habits and more mindful of their next meal. One participant said, “It (eTRIP) incentivizes me to stick to my diet plan because I am reminded of my diet plan daily. Ticking the box that indicates ‘I did not meet my diet plan’ made me guilty and it motivates me to opt for healthier food choice the next time round” (Female, Chinese, 22 years old). Another participant said, “I really liked the eTRIP app! Has a lot of potential for further expansion and use by more people. I like how it sends prompts during selected times of the day to be careful of what we see on social media. The rating of our mood before meals also helps me know how mood can affect my eating patterns. Lastly, the stopwatch function is great because it reminds me to eat more mindfully” (Male, Chinese, 27 years old).

#### Personalized Reminders With Prompts and Chatbot

Some participants mentioned the appreciation for reminders to check in with themselves in terms of the triggers of overeating. One participant said, “I like that there’s a reminder to check in for every meal and users get to decide what time the app should prompt!” (Female, Indian, 27 years old). Some also suggested to develop the prompting system to prompt based on the user’s previous check-in timings to optimize the prediction of mealtimes and prompt the check-in sessions intuitively. One participant suggested, “I think what would make this better is if you could aggregate the time the meals are entered from the past few days and estimate the time the user will normally eat and auto-adjust the timing…” (Female, Chinese, 23 years old). Others suggested to include reminders of how to make their meal options healthier, “Might be good to have reminders that reminds us to eat healthy with some tips on how to choose food” (Male, Chinese, 25 years old).

#### Food Logging With Image Recognition

Many participants highlighted their appreciation for the image recognition-based food logging, as it was accurate and convenient for food logging. One participant said, “It is very accurate in determining the food I’ve eaten just from the picture, and this saved me a lot of time from typing out the food I’ve eaten” (Male, Chinese, 21 years old).

#### Engaging With a Simple, Easy, and Appealing User Interface

All the participants who commented on the user experience expressed being impressed with the user interface and structure. One participant said, “the flow was smooth, quite clear. Graphics were cute. Very easy to input my info (information) especially from the homepage, I like how there’s the ability to skip a meal” (Female, Malay, 25 years old). Another participant said, “The app is very smart, … yes it’s very easy to fill and I loss (lost) like 0.5kg?” (Female, Chinese, 25 years old).

### Participants’ Suggestions

In terms of the areas for improvement, the participants preferred to have (1) more options and rating scales for each domain of eating trigger instead of typing out in the “others” field (although there was a stored text for repeated entries); (2) summary of the instances where one was able to achieve the goal of the day, which the user sets daily for the next day (based on a user preset list of goals); (3) examples of standard portions and frequency of meals; and (4) feedback on how to improve upon the unhealthy meals logged.

## Discussion

### Overview

Real-time interventions that can effectively address eating lapse triggers and improve eating behavior self-regulation, lapse events, weight loss, and weight maintenance remain unclear [45]. OnTrack, a just-in-time adaptive intervention that has been tested, is a smartphone app that uses machine learning to predict dietary lapses based on the repeated assessments of lapse triggers (ecological momentary assessment). OnTrack is used in conjunction with existing weight loss apps such as WeightWatchers app and provides personalized recommendations to prevent dietary lapses. The compliance rate for completing the lapse trigger survey in OnTrack was 62.9% over 3 months, and the studied sample was mostly females who were Whites [46]. Evidence has shown that factors influencing obesity and overweight are population-specific, influenced by socioeconomic, cultural, and genetic factors among others [45,47]. Singapore is a multiethnic society with a unique food culture influenced by various racial beliefs and traditions [48]. The differences in geographical, social, environmental, and genetic characteristics could define a different set of triggers and response to such weight loss apps.

### Principal Findings

In this paper, we report the effectiveness of a weeklong AI-assisted weight loss app for improving overeating habits, snacking habits, immediate thinking, self-regulation of eating habits, depression, and physical activity. Interestingly, there were no significant improvements in the anxiety symptoms before and after using eTRIP, potentially due to the already low level of anxiety in those who completed the program (ie, ceiling effect). We also report corresponding qualitative user feedback on the experience with using eTRIP, where the users appreciated the app for enabling them to become more mindful of self-monitoring; personalized reminders with prompts and chatbot; food logging with image recognition; and engaging with a simple, easy, and appealing user interface. The significant improvements observed among the participants in this study reveal the potential of this app to influence weight loss in the context of a Southeast Asian cohort with overweight and obesity. The qualitative feedback also informs future app development to enhance user engagement and reduce dropout rates.

Eating habits contribute to overweight and obesity [49,50]. Encouraged by an obesogenic environment, overeating is commonly triggered by situational factors such as food novelty or variety, social company (eg, eating with certain people), affect emotional states (which trigger emotional eating), and distractions (eg, concurrent tasks) [30,50-53]. Other studies have suggested that people at risk for obesity exhibit hyperresponsivity in the neural reward system to calorie-dense foods, which is associated with increased food consumption [54]. Alongside users’ feedback that the app made them more mindful of their eating patterns, the significant improvement in overeating and snacking habits could have been due to an increased awareness of one’ maladaptive eating habits and subsequently, the motivation to change. This coincides with a review that reported the effectiveness of mindful eating interventions on reducing food consumption in people with overweight and obesity [55]. Our qualitative findings showed that by self-monitoring one’s eating behavior through chatbot-initiated check-ins, one could enhance mindful eating and reduce overeating without the need for undergoing mindful eating training. This could eventually lead to a reduction in total food consumption and weight loss. However, more quantitative evidence is needed to support this point.

It is noteworthy that some participants reported skipping meals as planned, which might have led to reduced energy consumption. However, this has to be examined further, as studies have shown that the calories avoided during a skipped meal may be compensated by an increase in snacking or overeating during mealtimes [56]. One additional element that can be explored in future studies is the effectiveness of promoting healthy snacking, which includes snacking on foods rich in proteins, fruits, vegetables, and whole grains, as opposed to nutrient-poor and energy-dense foods [57,58]. These healthier alternatives have been found not only to be associated with earlier satiety but also to be more nutritious, with their contents being more consistent with the established dietary recommendations and guidelines [59,60]. This strategy can be explored in conjunction with the current approach to decrease participants’ overall snacking habits.

The improvement in the self-regulation of eating habits could be attributed to several factors, including the app content focused on reminding participants of their weight loss goals and to adopt healthier eating habits of less snacking and overeating during mealtimes. In particular, in commonly stigmatized populations like those with overweight and obesity, personalization of interventions enhances one’s feeling of being taken care of, nurtured, and respected, providing them with a sense of confidence [61]. This may have improved individuals’ willingness to engage with the eTRIP content, knowing that they would be well-respected and seen as individuals through personalized chatbot conversations and reminders [62]. Other studies have shown that personalized eHealth interventions are more effective than conventional programs in enhancing weight loss maintenance, BMI, waist circumference, and various other metabolic indicators [63].

In conjunction with improvements in eating habits, participants also engaged in greater levels of physical activity by the end of this study. Increased health consciousness and self-education about the impacts and types of physical activity are factors that may explain the observed increased level of physical activity among the participants [64]. Various studies have found that a combination of diet and exercise is superior to diet-only interventions in inducing weight loss [65]. The level of physical activity is also an important factor for improving long-term weight loss [66-68]. Moderate amounts of physical activity were observed to prevent weight regain after weight loss [65,68,69]. In addition, the American College of Sports Medicine recommends 200-300 minutes of moderate physical activity a week to prevent similar weight regain [70]. In our study, low and moderate levels of exercise were seen to increase significantly among the participants. Although the sustainability of the increase in the exercise levels is still unknown, the preliminary data are encouraging to show the potential of the app in impacting physical activity. The amounts of high levels of exercise were, however, not impacted significantly. Additional interventions, including the provision of educational materials about the benefits of and types of exercise, along with personalized reminders for exercise can potentially further increase the success of the app in increasing moderate and high levels of exercise among its participants [71,72].

In addition to improvements in the eating habits and levels of physical activity, there were changes in the psychological factors among the participants. The mean depressive symptoms were significantly decreased at the end of the weeklong program. Various studies have shown that healthy living characterized by various factors such as healthy eating and sufficient levels of physical activity have the potential to positively impact psychological factors such as mood and emotions [73]. Healthy eating with adherence to dietary recommendations has been found to reduce the levels of inflammation, increase the levels of various micronutrients such as vitamins, and regulate the levels of simple sugars, all of which are protective against mental illnesses, especially depression [73-76]. Studies have also shown that the use of chatbots in the app may decrease depressive symptoms among some participants. Chatbots provide individuals the ability to provide self-care in an environment that is neither costly nor stigmatizing [77]. This may enable participants to be more open with their emotions, as well as to have an outlet to gain relief through their interaction with the chatbot as a proxy of human interaction [77]. Studies have shown that improvements in psychological factors such as depressive symptoms have been positively associated with weight loss and maintenance, further increasing the effectiveness of weight loss efforts among participants with overweight and obesity [45].

### Strengths and Limitations

This study was the first to characterize the effectiveness of an AI-assisted weight loss app in the context of a Southeast Asian cohort. One strength of this study was the demographics of the participants, which was generally representative of the Singaporean population in terms of sex and ethnicity. The consideration of population-specific determinants of obesity and overweight during the design of the app would have also increased its applicability in this population [78], having considered the various nuances and practical needs of its target demographic. For example, the food image recognition system, which was built based on local food items, reduced the amount of time and effort required for the logging of food, improved the usability of the intervention, and enhanced user experience. The success of this app thus provides evidence that the consideration of population-specific underpinnings and practical requirements were essential toward the successful design and implementation of a weight loss intervention [79].

Although the app presents significant potential in this weeklong trial, this study is limited due to its short time frame. This presents with difficulties in understanding the midterm to long-term impacts of using the app. However, it is reassuring that despite the limited time frame of this study, the various behavioral and psychological indicators were observed to be significantly improved. Through the feedback gathered from the participants, the app may be improved in specific aspects, including (1) refining choices available for the various survey fields such as the provision of a drop-down menu for the selection of weight loss goals; (2) providing additional feedback and weekly summaries to the participants for knowledge of their progress in various aspects; and (3) providing educational materials to provide participants with the means to improve, especially for what to do after eating lapses and suggestions for healthy snacking. Another limitation was the lack of feedback quotes from older individuals as opposed to those from younger individuals. This could be due to various reasons, of which decreased media literacy among older individuals might present additional obstacles for the provision of feedback [80]. Lastly, we did not collect information on the participants’ medical and pharmacological history, where certain diseases and drugs are known to influence weight gain through various metabolic and neural pathways. Weight and waist circumference were also self-reported, and thus, data from these measures should be interpreted cautiously.

### Conclusion

This study was the first to characterize the effectiveness of an AI-assisted weight loss app in the context of a Southeast Asian cohort. The positive findings of this study show the feasibility of implementing this app and the large potential it has in impacting weight loss efforts, especially among individuals with overweight and obesity. Efforts should be made to lengthen and upscale this program for a greater understanding of the midterm to long-term effects of this app.

## Appendix

Multimedia Appendix 1 TREND (Transparent Reporting of Evaluations with Nonrandomized Designs) checklist.

Multimedia Appendix 2 Details on outcome measures.

## Abbreviations

AI: artificial intelligence
eTRIP: Eating Trigger-Response Inhibition Program
TREND: Transparent Reporting of Evaluations with Nonrandomized Designs
