# Correction: Effectiveness of an Artificial Intelligence-Assisted App for Improving Eating Behaviors: Mixed Methods Evaluation

**DOI:** 10.2196/62767

**Published:** 2024-06-07

**Authors:** Han Shi Jocelyn Chew, Nicholas WS Chew, Shaun Seh Ern Loong, Su Lin Lim, Wai San Wilson Tam, Yip Han Chin, Ariana M Chao, Georgios K Dimitriadis, Yujia Gao, Jimmy Bok Yan So, Asim Shabbir, Kee Yuan Ngiam

**Affiliations:** 1 Alice Lee Centre for Nursing Studies, Yong Loo Lin School of Medicine, National University of Singapore Singapore Singapore; 2 Department of Cardiology, National University Hospital Singapore Singapore; 3 Yong Loo Lin School of Medicine, National University of Singapore Singapore Singapore; 4 Department of Dietetics, National University Hospital Singapore Singapore; 5 School of Nursing, Johns Hopkins University Baltimore, MD United States; 6 Department of Endocrinology ASO/EASO COM, King's College Hospital NHS Foundation Trust London United Kingdom; 7 Division of Hepatobiliary & Pancreatic Surgery, Department of Surgery, National University Hospital Singapore Singapore; 8 Division of General Surgery (Upper Gastrointestinal Surgery), Department of Surgery, National University Hospital Singapore Singapore; 9 Division of Thyroid & Endocrine Surgery, Department of Surgery, National University Hospital Singapore Singapore

In “Effectiveness of an Artificial Intelligence-Assisted App for Improving Eating Behaviors: Mixed Methods Evaluation” (J Med Internet Res 2024;26:e46036) the authors noted one error.

The last name of author GKD:

Dimitriadish

Has been revised to:

Dimitriadis

The correction will appear in the online version of the paper on the JMIR Publications website on June 7, 2024, together with the publication of this correction notice. Because this was made after submission to PubMed, PubMed Central, and other full-text repositories, the corrected article has also been resubmitted to those repositories.

