# Supplementary material for: Effectiveness of an Artificial Intelligence-Assisted App for Improving Eating Behaviors: Mixed Methods Evaluation
Source: J Med Internet Res. 2024 May 7;26:e46036. doi: 10.2196/46036 (PMC11109864; doi:10.2196/46036)
Supplement: Multimedia Appendix 2 [file jmir_v26i1e46036_app2.docx]

**Appendix 2:** Details on outcome measures (Manuscript under review)

Data was collected through the Qualtrics, an online survey platform.

***Sociodemographic and anthropometric profile***

Data on age, sex, marital status, race, religion, highest education level, employment, and per capita household income. BMI and waist circumference were self-reported during the recruitment survey and app onboarding.

***Intention to change eating behaviors***

Intention was measured using the questions, “I intend to change my eating behaviors to lose weight?” Responses were measured on a 7-point Likert scale (1 = Strongly Disagree; 2 = Disagree; 3 = Somewhat Disagree; 4 = Neutral; 5 = Somewhat Agree; 6 = Agree; 7 = Strongly Agree). [23]

***Self-regulation of eating behavior***

Self-regulation of eating behavior was measured on a five-point scale (1 = Never; 2 = Rarely ;3 Sometimes; 4 = Frequently; 5 = Always) using the five-item Self-regulation of Eating Behavior Questionnaire (SREBQ) (α=0.75). [24] It encompasses constructs such as “self-monitoring, appraising progress, … and the capacity to control behavior, thoughts and attention”. An example is “I’m good at resisting tempting food”. A mean score (three reverse coded items) of <2.8 represents low self-regulation, 2.8-3.6 represents moderate and >3.6 represents high self-regulation. It also includes a section on the food items that participants find most tempting. The validity of SREBQ had been established with a strong positive correlation with general measures of self-regulation, motivation and behavioral automaticity. [24] It has also been shown to be negatively correlated with food responsiveness and emotional overeating. [24]

***Consideration of future consequences***

Consideration of future consequences refers to the extent to which one considers future consequences of current behaviors and has been established as a cognitive motivational construct that influences behaviors related to energy balance. [25] It was measured on a seven-point scale using the 6-item Consideration of future consequences scale (CFCS-6). [26] It comprises of two subscales (immediate [reverse coded] and future) of which a higher sum average represents a more future-oriented thinking. Both subscales demonstrated good internal reliability (α_immediate_=.821; α_future_=.775). [25]

***Overeating habit***

Habit strength of overeating and snacking was measured on a seven-point scale using the 12-items Self-Report Habit Index (SRHI). [27] It consists of three factors namely automaticity; behavior frequency; and self-identity and is a commonly used toolto measure behaviors related to energy balance (e.g. physical activity & unhealthy diet). [28] SRHI has been shown to be reliable upon 1-week test-retest (α = 0.90; P<0.001) and valid when correlated with the response-frequency measure of habit (r = 0.58, p < .001). [27]

***Physical activity***

Physical activity over the past 7 days was measured using the seven-item International Physical Activity Questionnaire Short-Form (IPAQ-SF). The IPAQ-SF assessed duration, frequency, exertion level, and amount (vigorous, moderate, and walking) of physical activity. [29] Scores were transformed to Metabolic Equivalent Task (MET) by multiplying day and minute scores according to the guidelines (walking, moderate, and vigorous intensity activity corresponds to 3.3 METs, 4.0 METs, and 8.0 METs respectively). Scores were summed and classified into low (< 600 METmin/wk), moderate (≥ 600 METmin/wk) and high (≥ 3000 MET min/wk) physical activity. Its validity has been well-established with accelerometery. [30]

***Depression and anxiety***

Depression and anxiety were measured using the two-item Patient Health Questionnaire-2 (PHQ-2)[31] and two-item Generalized Anxiety Disorder-2 (GAD-2)[32] respectively. A four-point scale (1=not at all, 2=several days; 3=more than half the days; 4=nearly every day) was usd. Sum scores of ≥3 for each scale represented a possible case of depression and anxiety respectively.
